# Effectiveness of mHealth Interventions for Improving Contraceptive Use in Low- and Middle-Income Countries: A Systematic Review

**DOI:** 10.9745/GHSP-D-20-00069

**Published:** 2020-12-23

**Authors:** Banyar Aung, Jason W. Mitchell, Kathryn L. Braun

**Affiliations:** a Office of Public Health Studies, University of Hawai‘i at Mānoa, Honolulu, HI, USA.; b Access to Health Fund, United Nations Office for Project Services, Myanmar.; c Florida International University.

## Abstract

**Background:**

mHealth interventions are being tested to improve contraceptive uptake in low- and middle-income countries (LMICs); however, the effectiveness of these interventions has not been systematically reviewed.

**Objectives:**

The primary objective of this systematic review was to assess the effectiveness of mHealth interventions to improve contraceptive uptake and adherence in LMICs. A second objective was to identify mHealth features and behavior change communication components used in these mHealth interventions.

**Methods:**

A systematic search was conducted of online databases for peer-reviewed articles that reported on intervention studies with men and women from LMICs and measured mHealth intervention impact on contraceptive uptake and/or adherence. Key search terms included “mHealth” or “mobile health,” “contraception” or “family planning,” and “low- and middle-income countries.” PRISMA guidelines were followed for reporting review methods and findings. The Cochrane risk-of-bias 2 tool for randomized trials was used to assess the risk of bias of the included studies. The GRADE approach was used to determine the quality of evidence.

**Results:**

Eight randomized controlled trial studies met the inclusion criteria. Four studies experienced implementation challenges (e.g., intervention components were not utilized fully by participants, intervention participants did not receive the full intervention content, contamination, low response rate, and/or missing data). Only 3 interventions were found to be effective, and these included a “push” approach, interactive communication, information tailored to participants, motivational messaging, and male partner involvement.

**Conclusion:**

To date, the delivery of mHealth interventions for improving family planning in LMICs has met with implementation challenges that have reduced the researcher’s ability to test intervention effectiveness. Although 3 of 8 studies found improved contraceptive use in the intervention group, the review cannot draw concrete conclusions on the overall effectiveness of mHealth interventions to increase contraceptive use in LMICs. Further research with robust program fidelity is recommended.

## Abstract (precis)

Do mHealth interventions help reduce unmet contraceptive needs in low- and middle-income countries by attempting to increase the uptake of modern contraceptive methods? Which mHealth features and behavior change communication components were used in these mHealth interventions? This review aimed to answer these questions and assess the impact of these interventions on contraceptive uptake outcomes.

## Teaser

**Key Findings:**

- Of the 8 mHealth family planning interventions that met inclusion criteria, 3 studies improved family planning outcomes and 4 studies experienced implementation issues.
- Further research is needed to encourage robust program fidelity of mHealth family planning interventions, along with a more thorough understanding of what mHealth and behavior change components are needed to improve family planning outcomes in low- and middle-income countries.

**Key Implications:**

- A “push” approach, interactive communication, information tailored to participants, motivational messaging, and male partner involvement appear to be tied to better family planning outcomes.
- Program managers and researchers should consider improvements in protocols and fidelity that are needed to more accurately assess how well mHealth family planning interventions impact outcomes in low- and middle-income countries.

## INTRODUCTION

By the end of the Millennium Development Goals in 2015, the maternal mortality ratio had declined by 45% from 1990.^1^ Despite this progress, every day, 810 mothers—94% from low- and middle-income countries (LMICs)—continue to die from preventable causes associated with pregnancy and childbirth.^2^ To help reduce maternal deaths in LMICs, the Safe Motherhood Initiative outlined family planning as 1 of 6 “pillars” of safe motherhood.^3^ One viable solution to reduce maternal mortality in LMICs is to increase the uptake of contraceptives, which in turn will reduce the number of unwanted pregnancies.^4^ Fulfilling the current unmet need for contraceptives can help reduce maternal mortality by preventing 104,000 maternal deaths annually,^5^ while also helping to improve child survival rates by promoting the healthy timing and spacing of pregnancy.^6^ ^,^ ^7^

Unmet needs for family planning are attributed to insufficient knowledge and access to family planning services.^8^ Many women who want to avoid pregnancy do not use modern contraceptive methods due to limited or inaccurate knowledge about side effects of contraceptives or the misperception that conception is not possible while breastfeeding or during certain times of the menstrual cycle.^8^ In 2017, the United Nations reported that at least 1 in 10 married or in-union women had unmet family planning needs globally.^9^ Fulfilling women’s unmet contraceptive needs is an important global public health goal.^10^

Mobile phone ownership in LMICs has proliferated,^11^ providing new technologies to deliver educational and access-related information about reproductive health and family planning to hard-to-reach populations.^12^ The use of mobile technology in health care (i.e., mobile health or mHealth) has gained popularity globally and has been found to reduce health care costs, improve the quality of health care, and encourage prevention-related behaviors.^13^ In 2018, world governments unanimously adopted a World Health Assembly resolution calling on the World Health Organization (WHO) to develop a global digital health strategy to support countries’ efforts toward universal health coverage.^14^ Subsequently, WHO released the guideline *Recommendations on Digital Interventions for Health System Strengthening*, which endorses the use of mobile technology for targeted client messaging of health services in LMICs.^15^

As a platform, mHealth has been used to offer educational information about sexual and reproductive health, as well as the locations of family planning service providers.^16^ Additionally, mHealth affords individuals with fewer logistical barriers because they can quickly, conveniently, and confidentially seek information about family planning and related resources instead of having to go to a clinic or see a health care provider to obtain this same information.^17^ Several interventions have been implemented to assess whether mHealth technologies could be used to help reduce unmet contraceptive needs in LMICs by attempting to increase the uptake of modern contraceptive methods.^18^ ^–^ ^25^

Three published reviews^26^ ^–^ ^28^ explored the effectiveness of mHealth interventions for different contraceptive outcomes. Smith et al.^28^ assessed the effect of interventions delivered via mobile phone for improving contraceptive use in 5 randomized controlled trials (RCTs) conducted in the United States, Cambodia, and Israel. Only one of the studies occurred in an LMIC. The review concluded that interactive voice messages and communication with a counselor improved postabortion contraception, and the combination of unidirectional (i.e., one-way messages) and interactive daily educational text messages (i.e., back-and-forth) improved adherence in using oral contraceptives.

In another review, L'Engle et al.^26^ examined 35 studies that used mobile phones to improve adolescent sexual and reproductive health, inclusive of contraceptives. Only 3 of the 35 studies were from LMICs. The authors found evidence that including text messages in interventions may improve adolescent sexual health, but the information provided in the studies was insufficient for understanding, replicating, or scaling up mHealth interventions.

Rousseau et al.^27^ conducted a systematic review with 22 studies to explore the general impact of smartphone applications on contraceptive decision making and knowledge. Fifteen of the 22 studies were based in the United States, 3 were conducted in an LMIC, and the locations of the 4 remaining studies were not specified. The reviewers found that apps may be useful as aids to improve contraceptive use and prescription of contraception, but they were not reliable sources of information. The authors noted that the quality of the studies was heterogeneous, adding to the difficulty in drawing conclusions about the impact of mHealth apps on contraceptive knowledge and usage.

Although previous systematic reviews assessed the effectiveness of mHealth interventions for family planning, only the review by Smith and colleagues^28^ focused exclusively on contraceptive uptake, while other 2 systematic reviews involved other outcomes (e.g., contraceptive knowledge). Furthermore, only 7 studies included in these 3 reviews were based in an LMIC, and only 1 measured contraceptive use. In sum, the bulk of research involving mHealth on family planning and uptake of modern contraceptives has occurred in higher-income countries, with few trials and studies having occurred in LMICs. Given the disparities of maternal mortality and unmet family planning needs in LMICs, a more thorough examination of the role of mHealth in improving the uptake of modern contraceptives in LMICs is needed.

Most mHealth research on family planning and uptake of modern contraceptives has occurred in higher-income countries, with few trials and studies in LMICs.

The primary objective of the present systematic review was to assess the effectiveness of mHealth interventions in improving contraceptive uptake in LMICs. The secondary objective of the systematic review was to identify which mHealth features and behavior change communication (BCC) components were used in the mHealth interventions that occurred in LMICs.

## METHODS

Review findings are reported based on the Preferred Reporting Items for Systematic Reviews and Meta-Analyses-Protocols (PRISMA) guidelines.^29^ The review protocol was preregistered in the PROSPERO database (CRD42020153409).

### Inclusion Criteria

#### Type of Studies

Experimental studies that evaluated the intervention effectiveness through RCTs and nonrandomized interventional studies were considered for the review.

#### Type of Participants

Women and men from LMICs, as classified by the World Bank,^30^ were included. The WHO definition of women of reproductive age (15–49 years old)^31^ was not used because more than 1 in 3 (about 250 million) girls were married or in union before age 15, with the highest rates found in LMICs in South Asia and sub-Saharan Africa.^32^ Postpartum and postabortion women were also included.

#### Type of Interventions

We included studies in which the intervention was delivered using any form of mHealth such as mobile apps, messaging platforms or short messaging system (SMS), telephone calls, or geolocational features (e.g., GPS or Global Positioning System). We included the interventions that sought to improve contraceptive uptake and/or adherence compared with standard care or another intervention. mHealth interventions were identified based on the definition of the WHO Global Observatory for eHealth.^33^

We focused on interventions that sought to improve contraceptive uptake and/or adherence compared with standard care or another intervention.

#### Type of Outcome Measures

For the purposes of this review, we included the outcome measurement of uptake or adherence to any modern contraceptives^34^ including permanent methods (female sterilization and vasectomy); long-acting reversible contraceptives (implants and intrauterine devices); and shorter-acting contraceptives (injectables, pills, male and female condoms, diaphragms, spermicides, and cervical caps). We acknowledge that other nonbiomedical methods such as fertility awareness methods and withdrawal methods exist, but these were not included in our definition. We accepted whichever method by which the outcome was assessed in the included mHealth intervention trials/studies, including by self-report through surveys. Interventions were included even if the uptake and/or adherence to contraception was not the primary outcome measured or was measured in conjunction with other contraceptive outcomes such as knowledge of contraception.

### Search Strategy

The search was conducted by BA in July 2019. A filter was set to include articles from 2005, since mobile subscriptions reached 23% of populations in LMICs in 2005 (compared with only 4% in 2000).^35^ PubMed, Web of Science, EBSCOhost, CINAHL, and The Cochrane Library were searched. Key search terms used were “intervention*”; “program*”; “mHealth”; “mobile health”; “telemedicine”; “cell phone*”; “SMS”; “apps”; “contraception”; “contraceptive*”; “family planning*”; “birth spacing”; “developing countr*”; and “low and middle income countr*”. LMICs were further searched by detailing regions such as Africa, Asia, Pacific Islands, South America, Central America, Latin America, Eastern Europe, and Central Asia. Reference lists of identified articles were searched. We retrieved study protocols of included studies and assessed method details. We contacted authors of included studies if the study protocol was not published and when additional information was needed. Only articles in English were included due to the reviewers’ language limitation.

### Data Collection and Analysis

The search was completed by BA and KLB independently. Duplicates were removed and titles and abstracts were assessed applying the inclusion criteria. Screened articles were read in full, again, by applying the inclusion criteria. Discrepancies were resolved by discussion.

BA and KLB extracted information from the studies, including the country in which the study was conducted, intervention details (e.g., mHealth features, mode of delivery, BCC components, frequency, duration), participant characteristics (age, gender, postabortion, postpartum, etc.), sample size, study design, and outcome(s) relative to modern contraceptive use. Microsoft Excel was used to store and organize the extracted data from included studies.

mHealth features and BCC components of interventions were extracted and categorized by BA and JWM into telephone-based, text/SMS, and apps; communication pathway (unidirectional or interactive); how family planning information was delivered (“push” telephone service, “push” messaging service, or “pull” messaging service); and additional intervention components (motivational message, tailored information, partner counseling, searching for the nearest service provider, “role model” stories, and intervention delivered via health workers). Push approaches referred to the delivery of the intervention (family planning information) at predefined intervals or frequencies, while pull approaches relied on the consumer searching for information without being prompted. mHealth features and BCC components were analyzed against contraceptive use or adherence outcome.

### Assessment of Study Quality and Risk of Bias

Quality assessment of the included studies was done according to the revised Cochrane risk-of-bias tool for randomized trials (RoB 2), as only RCTs that met the inclusion criteria were included in the review.^36^ We examined 5 bias domains of RoB 2: randomization process; deviations from intended intervention; missing outcome data; measurement of the outcome; and selection of the reported results. The risk-of-bias judgments for each domain were “low” or “high” risk of bias or “some concerns.” Risk of bias was assessed based on the effect of assignment to intervention, the “intention-to-treat” effect, for the included studies. BA and JWM individually and separately assessed risk of bias for quality before comparing notes for each included study.

### Measures of Treatment Effect

We planned to determine risk ratios, as measures of treatment effect, for dichotomous outcomes, and mean differences for continuous outcomes, with 95% confidence intervals. However, we were unable to obtain adequate data from included studies to determine effect sizes.

### Assessment of Heterogeneity

We did not conduct a meta-analysis due to the diversity of intervention components and outcome measures that were used in the included studies. However, clinical heterogeneity (i.e., variability in participants, interventions, outcomes studied) and methodological heterogeneity (i.e., variability in study design and risk of bias) of the included studies were characterized.

### Assessment of Publication Bias

We were unable to execute a funnel plot to identify the publication bias due to the diversity of intervention components and outcome measures that were used in the included studies.

### Data Synthesis

We conducted the analysis according to the guidelines specified in the *Cochrane Handbook for Systematic Reviews of Interventions*.^37^ Quality of evidence for included studies was assessed using the Grading of Recommendations Assessment, Development and Evaluation Working Group (GRADE) approach.^38^ RCTs were considered high quality and were downgraded by 1 level for “serious” (or 2 levels for “very serious”) risk of bias; unexplained heterogeneity; indirectness of evidence; imprecision of effect estimates; or publication bias.

## RESULTS

**FIGURE 1.**
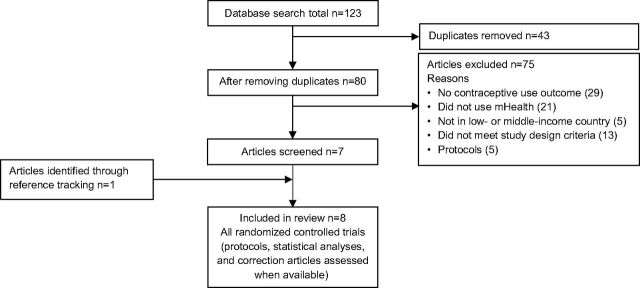
PRISMA Flow Diagram for the Systematic Review of Experimental Studies Evaluating the Effectiveness of mHealth Interventions on Contraceptive Uptake in Low- and Middle-Income Countries Abbreviation: PRISMA, Preferred Reporting Items for Systematic Reviews and Meta-Analyses-Protocols.

Figure 1 shows the PRISMA flow diagram for the systematic review. Among the 123 publications identified in the database search, 43 duplicates were removed and 80 studies were assessed; all 80 studies were published in English. After titles and abstracts were screened, 73 studies were excluded for not meeting inclusion criteria and 7 articles were further assessed. One additional article was identified through reference tracing. Eight studies met the inclusion criteria for this systematic review.

### Study Characteristics

Of the 8 studies included in this review, 3 were conducted in Kenya,^18^ ^,^ ^23^ ^,^ ^25^ 1 in Cambodia,^22^ 1 in Ecuador,^19^ 1 in Tajikistan,^20^ 1 in Palestine,^21^ and 1 in Bangladesh^24^ (Table 1). All 8 studies were parallel-group RCTs with 1:1 allocation, including a feasibility study with a small sample size.^24^ Study settings varied. Some were conducted in urban^18^ or peri-urban and rural areas,^22^ while others were conducted in a hospital or clinic setting^19^ ^,^ ^23^ ^–^ ^25^; settings for 2 studies were not specified.^20^ ^,^ ^21^ The studies also varied by types of participants: postpartum mothers,^19^ ^,^ ^23^ ^,^ ^25^ postabortion women,^22^ ^,^ ^24^ young people,^20^ ^,^ ^21^ and general public.^18^ Outcomes for 6 of the 8 studies were about contraceptive use and knowledge^18^ ^,^ ^20^ ^–^ ^22^ ^,^ ^24^ ^,^ ^25^; the other 2 studies measured the same outcomes and other maternal and child health indicators (e.g., exclusive breastfeeding and immunization coverage).^19^ ^,^ ^23^ Three of the 8 studies provided a description about the use of behavior change theory.^20^ ^,^ ^21^ ^,^ ^25^

**TABLE 1.** Summary of Studies Included in Systematic Review of mHealth Interventions Assessing Contraceptive Uptake in Low- and Middle-Income Countries, N=8

Authors	Country	mHealth Delivery Mode	Target Population	Study Design	Sample Size^a^ (Intervention/Control)	Frequency and Duration	Posttest and Follow-Up
Johnson et al.^18^	Kenya	HE via text messaging, “role model” stories, clinic database	General public	RCT (probably unblinded)^b^	13,629 (6,817/6,812)	Over 3 months	24 hours, 6 days, 3 months postenrollment
Maslowsky et al.^19^	Ecuador	Telephone-delivered HE and telephone access to a nurse	Postpartum women	Unblinded RCT	178 (102/76)	Within 48 hours of hospital discharge. Access to a nurse on-call during the first 30 days of the newborn’s life	3 months after delivery
McCarthy et al.^20^	Tajikistan	HE via app instant messaging	Young people (16–24), both genders	Single-blinded RCT	543 (275/298)	0–3 messages per day over 4 months	4 months after baseline
McCarthy et al.^21^	Palestine	HE via text messaging	Young women (18–24)	Single-blinded RCT	578 (289/289)	0–3 messages per day over 4 months	4 months after baseline
Smith et al.^22^	Cambodia	Voice messages and phone calls	Women, postabortion	Single-blinded RCT	300 (249/251)	6 automated voice messages ± telephone counseling within 3-month period	4 and 12 months postabortion
Unger et al.^23^	Kenya	HE via text messaging	Postpartum women	3-arm, unblinded RCT	300 (100/100/100)	Weekly until 12 weeks postpartum	From antenatal care attendance and followed through 10, 16, 24 weeks postpartum
Biswas et al.^24^	Bangladesh	HE via text messaging	Women, postabortion	RCT (probably unblinded)^b^	120 (60 /60)	Method-specific reminders/intervals (daily/weekly)	4 months postabortion
Harrington et al.^25^	Kenya	HE via text messaging	Postpartum women	Unblinded RCT	254 (125/129)	Weekly from enrollment to 6 months postpartum	6 months postpartum

Abbreviations: HE, health education (contraceptive information); RCT, randomized control trial; app, mobile application.

a Data from participants who were analyzed.

b Authors did not mention about blinding. This information was deduced from reading the studies.

All 8 studies were parallel-group RCTs with 1:1 allocation, but they varied in terms of setting, participants, outcomes, and theory.

### mHealth Features

mHealth features used in the 8 included studies varied (Table 2). Two studies used telephone calls. Smith et al.^22^ delivered interactive voice messages and provided counselor support via telephone calls upon participants’ request through the messages. Counselor phone support involved tailored information a range of contraceptive methods and motivation about using contraception, as well as helping participants in their search for family planning clinics. In contrast, the telephone call was made by a nurse to deliver health education about family planning in the study by Maslowsky et al.^19^

**TABLE 2.** mHealth Features and Behavior Change Communication Intervention Components Used in Studies Reviewed to Assess Effectiveness of Interventions on Contraceptive Uptake, N=8

Authors	Communication Pathway		Family Planning Information Delivery			Additional Intervention Components					Theory Framework Used	Frequency and Duration	Evidence of Effect (Improved Contraceptive Use)
	Unidirectional	Interactive	“Push” via Telephone	“Push” via Messaging	“Pull” via Messaging	Motivational Message	Tailored Information	Partner Involvement	Searching for Nearest Service Provider	Role Model Stories			
Johnson et al.^18^	**✓**				**✓**				**✓**	**✓**		Once	No^a^
Maslowsky et al.^19^	**✓**	**✓**	**✓**				**✓**					Once	No^b^
McCarthy et al.^20^	**✓**			**✓**		**✓**	**✓**				**✓**	0–3 messages per day for 4 months	No^c^
McCarthy et al.^21^	**✓**			**✓**		**✓**	**✓**				**✓**	0–3 messages per day for 4 months	No^d^
Smith et al.^22^	**✓**	**✓**	**✓**			**✓**	**✓**	**✓**	**✓**			2 times per month for 3 months	Yes^e^
Unger et al.^23^	**✓**	**✓**		**✓**		**✓**	**✓**					Weekly	Yes^f^
Biswas et al.^24^	**✓**			**✓**			✓*					Method-specific reminders (daily/weekly)	No^g,^ ^h^
Harrington et al.^25^	**✓**	**✓**		**✓**			**✓**	**✓**			**✓**	Weekly	Yes^i^

a Application was installed, and consumer received surveys for outcome measurement but had to search app for intervention materials. Recipients of full app showed increased knowledge over recipients of the limited app but no difference in contraceptive use.

b Participants received 1 phone-based educational session and were invited to call back for more education and counseling, but only 3 did. Intervention participants reported higher rates of breastfeeding and use of implants, but no differences were seen in overall contraceptive use.

c No statistically significant difference in contraceptive use and acceptability between intervention and control. Serious contamination occurred, and both the intervention and control participants received intervention messages.

d No statistically significant difference between the intervention and control groups in the use of effective contraception at 4 months. Intervention participants were more likely to find at least 1 method of effective contraception acceptable and had a higher mean knowledge score.

e Participants received 6 automated, interactive voice messages with counselor phone support, if they opted, and outcome was measured at 4 months and 12 months postabortion. Intervention group showed higher contraceptive use than the control group at both 4 months and 12 months, but the difference was only significant at 4 months.

f Both unidirectional and interactive short message service (SMS) interventions improved early postpartum contraceptive use over the control condition.

g Simple SMS reminder intervention did not improve contraceptive use at 4 months postabortion.

h Method-specific text message reminders to use method selected by participants, in their preferred language for the messages, including Bangla (Unicode), English, or phonetic Bangla in English fonts.

i The primary outcome of highly effective contractive (with less than 10% of failure rate) use at 6 months postpartum was significantly higher among women in the 2-way SMS group (69.9%) than in the control group (57.4%). Automated SMS text contained health education message and ended with actionable advice or a question to promote engagement.

Six studies used text messages as their primary mHealth feature to deliver health education and motivational messages about family planning.^18^ ^,^ ^20^ ^,^ ^21^ ^,^ ^23^ ^–^ ^25^ McCarthy et al.^20^ included an app in their intervention in Tajikistan to mainly deliver one-way text messages about contraception, common beliefs on family planning, and encouragement to use family planning. A similar intervention content was delivered via one-way text messages (without an app) to participants in the study conducted in Palestine by McCarthy et al.^21^ Using a text messaging platform named m4RH, Johnson and colleagues delivered information about family planning, a searchable database of clinics providing family planning services, and an optional role model stories feature.^18^

Mobile SMS delivery platform Mobile WACh and its variant Mobile WACh XY (with male partner involvement) were used by Unger et al.^23^ and Harrington et al.,^25^ respectively, to provide interactive intervention contents tailored to participant needs. The intervention tested by Biswas et al.,^24^ in Bangladesh, was a feasibility study conducted with a small sample size and it found no effect. Method-specific text message reminders were sent to participants about their select methods. It only involved unidirectional SMS reminders without other BCC components. However, the study found an mHealth contraceptive intervention was feasible, citing positive user engagement and participant acceptability.

Interactive communication was used in 4 studies.^19^ ^,^ ^22^ ^,^ ^23^ ^,^ ^25^ Seven studies used a push approach whereas only 1 study used a pull approach^18^ to deliver intervention content to participants. Of the 3 studies that reported improving contraceptive uptake,^22^ ^,^ ^23^ ^,^ ^25^ all used a push approach to deliver information and an interactive type communication.

The 3 studies that reported improving contraceptive uptake used a “push” approach to deliver information and an interactive type communication.

### BCC Components

Interventions utilized different intervention components to facilitate behavior change (Table 2), ranging from motivation to use family planning,^20^ ^–^ ^23^ tailoring of information,^19^ ^–^ ^23^ partner involvement,^22^ ^,^ ^25^ service provider search features,^18^ ^,^ ^22^ and role model stories.^18^ Two of the 3 interventions that reported improved contraceptive uptake included the involvement of a voluntary male partner.^22^ ^,^ ^25^ The study by Smith et al.^22^ involved counselor phone support that was tailored to the participant’s need and provided motivation to use postabortion contraception and information on nearest service providers.

The intervention by Unger et al.^23^ provided weekly unidirectional (partial intervention) and interactive (full intervention) family planning related educational and motivational SMS tailored to the recipient, and found that both full and partial interventions improved early postpartum contraceptive use over the control condition. The study by Harrington et al.^25^ used a variant of the intervention used by Unger et al.^23^ (Mobile WACh), but with a voluntary male partner involvement (Mobile WACh XY). However, male involvement did not have a significant effect on contraceptive use outcomes compared with having women as the only participants. In terms of frequency of intervention delivery, findings from these studies suggest that improved contraceptive use was associated with weekly^23^ or biweekly^22^ messaging rather than daily or a one-time delivery.

### Study Quality and Risk of Bias: Cochrane’s RoB 2 Tool

#### Randomization Process

**FIGURE 2.**
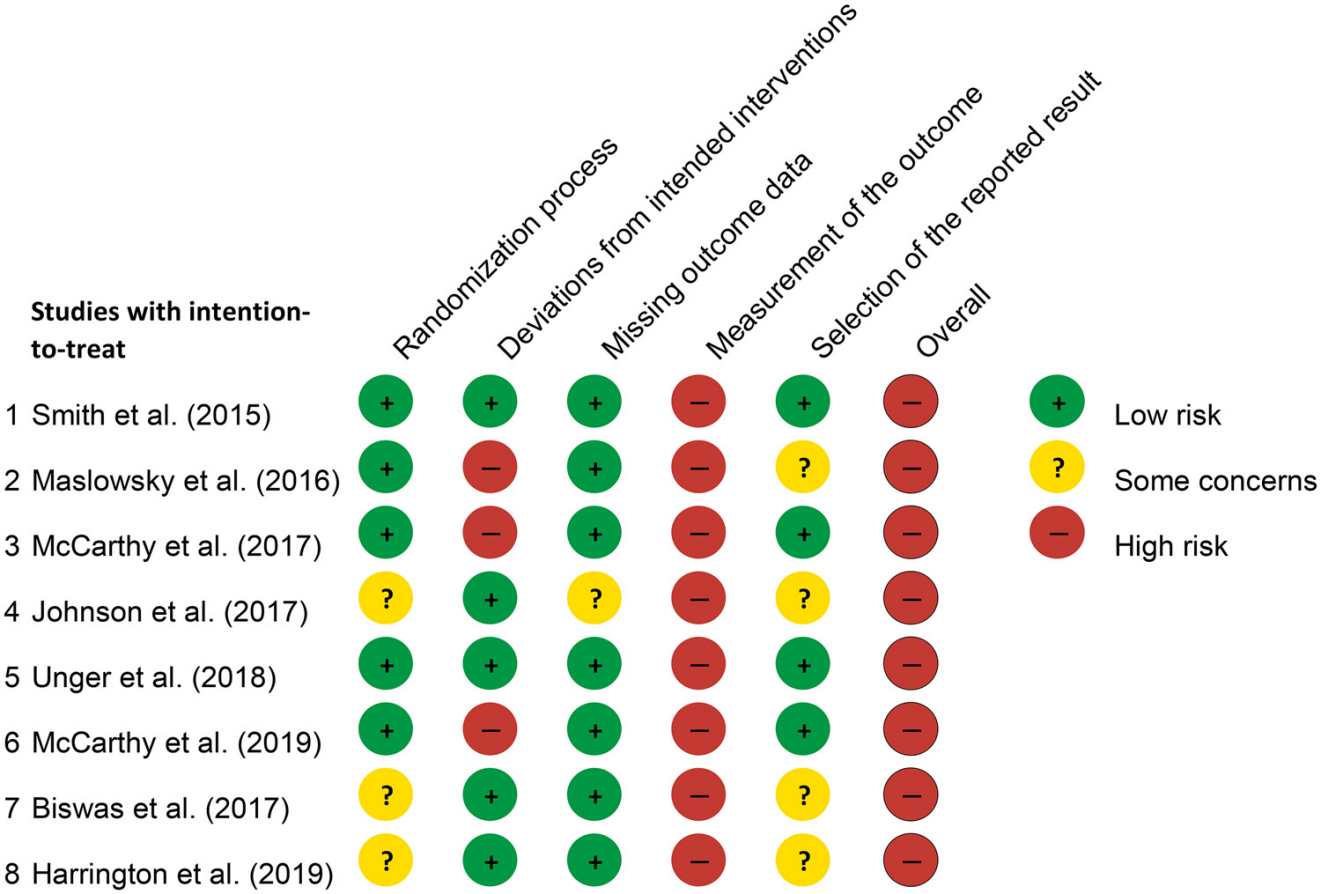
Risk of Bias in Studies of mHealth Interventions to Increase Contraceptive Uptake in Low- and Middle-Income Countries, N=8

Cochrane’s RoB 2 tool (Figure 2) classified 5 studies as low risk and 3 studies with some concerns for risk of bias in the randomization process domain.^18^ ^,^ ^24^ ^,^ ^25^ Johnson and colleagues^18^ used the alternation method of allocating participants to intervention and control groups, instead of true randomization. The trials by Harrington et al.^25^ and Biswas et al.^24^ had important baseline differences between their control and intervention groups.

#### Deviation From Intended Interventions

Five studies were conducted as intended and were thereby classified as having a low risk of bias (Figure 2).^18^ ^,^ ^22^ ^–^ ^25^ However, 3 studies deviated from their original protocol, resulting in a high risk of bias for this domain.^19^ ^–^ ^21^ The intervention by Maslowsky et al.^19^ had 2 parts, yet only 1 was delivered. It also had substantial contamination, with controls also receiving the intervention, as occurred in the intervention of McCarthy et al.^20^ In another study, participants did not receive the complete intervention.^21^

#### Missing Outcome Data

Seven studies had low risk of bias for missing outcome data (Figure 2). The study by Johnson et al.^18^ had some concerns for risk of bias due to having low retention rates: 20.9% of intervention and 21.3% of control participants were lost to follow-up for surveys that measured contraceptive uptake. To overcome this problem, researchers used multiple imputation methods for both groups but some concerns remain for risk of bias in this domain.^18^

#### Measurement of Outcome

All 8 studies had high risk of bias for measurement of the outcome (Figure 2). All the studies relied on self-reported outcomes obtained from final assessments; thus, the assessors were the participants and the outcome measurement may have been subjected to social desirability bias. The collection of outcome data was not blinded.

#### Selection of the Reported Result

As shown in Figure 2, half of the studies were at low risk for selective outcome reporting since all outcomes were reported in their results.^20^ ^–^ ^23^ The other half of studies had some concerns for selection of the reported result because the protocols containing details about their prespecified analytic plan were not published.^18^ ^,^ ^19^ ^,^ ^24^ ^,^ ^25^

#### Overall Risk of Bias

**FIGURE 3.**
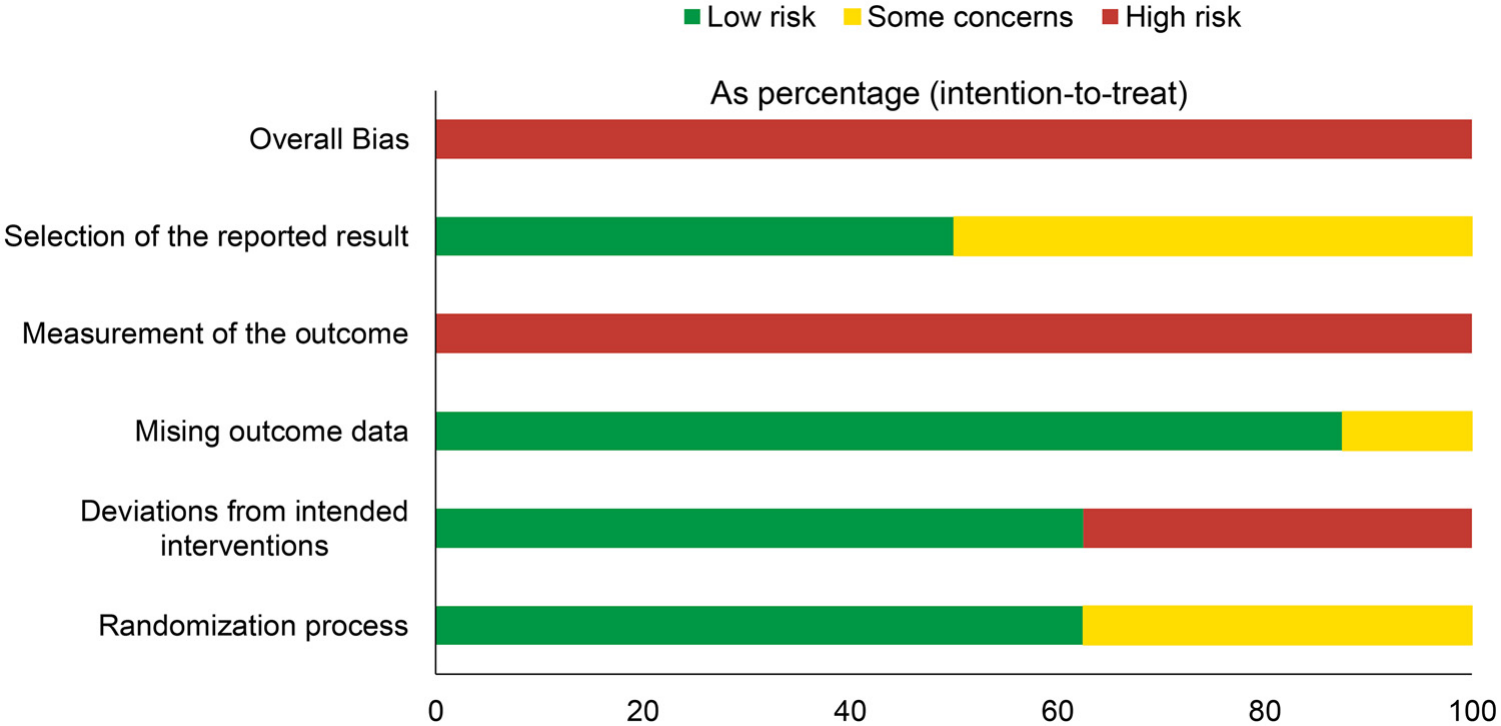
Summary of Risk of Bias of Studies of mHealth Interventions to Increase Contraceptive Uptake in Low- and Middle-Income Countries, N=8

Cochrane’s RoB 2 tool classifies the overall risk of bias to be considered high risk if any of individual domains (e.g., randomization process, missing outcome data) assessed were deemed high risk.^36^ As a result, all 8 studies were labeled as having an overall high risk of bias. Figure 3 provides a summary of the risk-of-bias assessment for the 8 included studies.

## DISCUSSION

### Summary of Findings

To our knowledge, this systematic review is the first to assess the effectiveness of mHealth interventions toward increasing contraceptive use in LMICs. Other systematic reviews have examined mHealth in family planning interventions, but few included studies from LMICs. Additionally, 7 of the 8 studies in the present review were not assessed in previous systematic reviews. Findings from the current systematic review reveal new information about the role that mHealth and BCC components have in improving contraceptive use in LMICs.

The current systematic review reveals new information about the role of mHealth and BCC components in improving contraceptive use in LMICs.

Of the 8 included studies, 3 reported improvements in family planning outcomes among people who received the intervention compared with controls.^22^ ^,^ ^23^ ^,^ ^25^ With respect to mHealth, 2 of the 3 studies used text messages,^23^ ^,^ ^25^ while the other study used voice messages and telephone counseling, which included information about the nearest family planning service provider.^22^ Two common traits that the 3 studies shared were the use of interactive communication and a push approach to deliver tailored intervention content to participants. Other commonalities were the use of motivational messages^22^ ^,^ ^23^ and the involvement of a male partner in the intervention.^22^ ^,^ ^25^

Given that only 3 of the 8 studies found improvements in family planning outcomes, the full extent that mHealth contributed to improvements in the use of modern contraceptives among participants cannot be determined. It is possible that certain types of mHealth features may be more advantageous to effect change in the use of modern contraceptives. For example, interactive communication and the use of a push approach to deliver intervention content entails engagement with participants. The frequency that the intervention information is delivered in studies that used the push approach may also have an impact on participants’ use of modern contraceptives. Some studies in this review delivered intervention information once,^18^ ^,^ ^19^ daily,^20^ ^,^ ^21^ weekly,^23^ ^,^ ^25^ or biweekly.^22^ Positive changes in outcomes were found in studies that delivered the intervention information on a weekly or biweekly basis, suggesting too frequent delivery may not resonate with participants with respect to their family planning needs.

Analysis of the BCC components used among the 8 included studies suggests tailoring information to the participant^19^ ^–^ ^23^ ^,^ ^25^ and potentially the use of motivational messages^20^ ^–^ ^23^ and/or the involvement of a male partner^22^ ^,^ ^25^ may play a role in improving contraceptive use. Among the 3 studies that showed significant improvements in outcomes (intervention vs. control), all tailored the information delivered, whereas 2 of the studies used motivational messages^22^ ^,^ ^23^ and 2 involved the male partners of participants.^22^ ^,^ ^25^ However, Harrington et al.^25^ conducted a subgroup analysis and found no significant differences in contraceptive use between participants who had their male partner enrolled versus those who did not.

### Comparison With Existing Literature

Our review found that interventions that showed significant improvement in contraceptive uptake used a combination of unidirectional and interactive communication styles and involved multiple BCC components. Notably, simple unidirectional text message reminders had no effect on improving contraceptive uptake. Such findings are consistent with the evidence from the systematic review that assessed the effect of mHealth interventions to improve contraceptive uptake, with 80% of studies involved having been conducted in developed countries.^28^

Interventions improving contraceptive uptake combined unidirectional and interactive communication styles and used multiple BCC components.

The International Conference on Population and Development set the involvement of men in family planning as a priority area.^39^ Smith et al.^22^ provided male partner telephone counseling by a nurse, upon request of the participant, and this component may have been a contributing factor in improving contraceptive uptake. Findings from prior studies support this possible explanation.^40^ ^–^ ^44^ For example, a case study spanning 5 generations of a family in an LMIC setting found that male involvement in family planning was associated with fertility decline in the family (due to increased use of contraception) and resulted in long-term benefits for women.^43^ In another study, Tao et al.^44^ found that involvement of the male partner in family planning decision making improved family planning knowledge and contraceptive continuation. Moreover, a systematic review that examined different BCC techniques used to improve contraceptive use in LMICs found that the most effective interventions were those that involved male partners.^45^ Prior research suggests the involvement of male partners is advantageous for family planning and the uptake of contraceptive methods. However, future research is warranted to assess whether the type of male partners differs (e.g., sexual/romantic relationship, family, friend), as well as the amount and frequency of their involvement toward achieving these outcomes.

Only 3 studies included in this review reported using a behavioral change theory.^20^ ^,^ ^21^ ^,^ ^25^ Two of them were conducted by the same researchers, who used the Integrated Behavioral Model,^20^ ^,^ ^21^ and the other study used the Theory of Planned Behavior.^25^ They are similar derivative theories of general behavioral prediction, with the most important determinant being motivation or intention as the interventions targeted. A systematic review by Cho et al.^46^ examined the use of theories in mHealth behavior change interventions conducted in the LMICs and also found that about one-third (5 of 14) of their included studies were based on a behavioral change theory. Well-tested behavioral change theories are useful to help guide the design and implementation of family planning interventions and programs.^46^ ^–^ ^49^ As the effectiveness of mHealth in family planning interventions in LMICs remains inconclusive, future research that uses behavioral change theory for contraception uptake is warranted and needed to help identify which intervention components (mHealth and behavior change) work best for family planning and why.

Systematic reviews on behavior change interventions of other health topics that used mHealth recommended the inclusion of certain components to increase the effectiveness of the intervention. For example, a systematic review on technologically driven weight-loss interventions by Khaylis et al.^50^ identified the following components as essential for improving outcomes: use of behavior change theory, self-monitoring, counselor feedback and communication, social support (motivation), and tailoring information. A meta-analysis by Webb et al.^49^ recommended that technology-based interventions make extensive use of theory, incorporate more BCC techniques, and use SMS or text messages to effectively promote behavior change. These reviews, along with the present one, suggest that the use of behavioral change theories is important to improve targeted behaviors, while also recognizing that further investigation is warranted to decipher which mHealth and BCC components and in what combinations lead to better family planning outcomes.

Behavioral change theories are important in improving targeted behaviors, and further investigation will identify which mHealth and BCC components lead to better outcomes.

### Considerations of Intervention Fidelity, Missing Data, and Limited Use

As noted in the risk-of-bias assessments, some studies included in this review reported issues with intervention fidelity or missing data. Findings from this review found important shortcomings in the included interventions that may have affected the study’s findings. Four out of 5 studies that did not find any significant changes in outcomes between trial arms had poor implementation or retention issues.^18^ ^–^ ^21^

Regarding fidelity, the study conducted in Tajikistan by McCarthy et al.,^20^ found contamination between trial arms (i.e., some controls received a portion of the intervention content) because of a misunderstanding between research partners. As such, the trial was assessed as the full intervention versus the partial intervention, instead of what was originally planned (i.e., comparing between intervention and control). Another study by McCarthy and colleagues^21^ conducted in Palestine had technical problems with the messaging platform used, which resulted in 60% of the intervention participants not receiving the full intervention. The outcomes measured were only based on the effect of partial receipt of the intervention versus the control. Further, contamination may have also occurred: 17% (39/235) of the control participants reported reading messages for someone else in the study and 17% (40/229) of intervention participants said that someone else in the study read their messages.

With respect to missing data, the intervention tested by Johnson et al.^18^ offered new users the m4RH app with text-message-based family planning information as well as a searchable database of service providers, with an option to receive role model stories of current users. The study had low response rates to its 3 assessments (range: 51.8% to 13.5%), and the proportion of participants who responded to more than 1 assessment was low. This large number of missing longitudinal data affected the statistical power for the study analyses, which may have influenced their findings.^18^

Not all participants will use all parts of an intervention. For example, Maslowsky et al.^19^ designed a 2-part intervention, with part 1 consisting of a one-time telephone-delivered health education session and part 2 consisted of having access to an on-call nurse for personalized advice (via telephone). Of the 178 study participants, only 3 participants used part 2; participants had to take the initiative to use part 2. Access to the on-call nurse for personalized advice included motivational support and tailored information, including where to receive contraceptive services. Numerous reasons may exist for why part 2 of the intervention was not used by the study participants and how its use and nonuse may have impacted the study’s findings. Future mHealth interventions for family planning ought to integrate monitoring of intervention delivery and other process evaluation techniques, as well as brief qualitative exit interviews or quantitative measures (e.g., Health-ITUES),^51^ to better understand the reasons why participants use and do not use certain parts of an intervention and how their usage affects the study’s findings.

Successful intervention outcomes necessitate well-implemented programs, and implementation fidelity is crucial for the intervention effectiveness.^52^ Half of the studies included in this review reported poor implementation or retention issues, which limits the ability to fully evaluate the intervention and assess its impact on contraceptive uptake outcomes. Future mHealth family planning trials ought to implement steps to help ensure the fidelity to the protocol and design of the intervention.

### Quality of the Evidence

Quality of the evidence was assessed using the GRADE approach^38^ (Table 3). Five trials were downgraded by 1 level under the domain of limitations in design and execution because they both had a high risk of bias in the measurement of the outcome.^22^ ^,^ ^23^ Under the same domain, 3 trials^19^ ^–^ ^21^ were downgraded by 2 levels due to high risk of bias from deviations from intended intervention, in addition to high risk of bias in measurement of outcome. Two trials were downgraded by 1 level under the imprecision of results domain due to small sample sizes.^19^ ^,^ ^24^ Overall, the quality of evidence was graded as moderate in 4 trials, low in 3 trials, and very low in 1 trial.

**TABLE 3.** Quality of Evidence of the Contraceptive Uptake Outcome Using the GRADE Approach in Studies Included in the Review, N=8

Study	Limitations of Detailed Design and Execution (Risk of Bias)	Unexplained Heterogeneity or Inconsistency of Results	Indirectness of Evidence	Imprecisions of Results	Publication Bias	Quality of Evidence
Smith et al.^22^	−1					⊕⊕⊕⊝Moderate
Maslowsky et al.^19^	−2			−1		⊕⊝⊝⊝Very low
McCarthy et al.^20^	−2					⊕⊕⊝⊝ Low
Johnson et al.^18^	−1					⊕⊕⊕⊝ Moderate
Unger et al.^23^	−1					⊕⊕⊕⊝ Moderate
McCarthy et al.^21^	−2					⊕⊕⊝⊝ Low
Biswas et al.^24^	−1			−1		⊕⊕⊝⊝ Low
Harrington et al.^25^	−1					⊕⊕⊕⊝ Moderate

Randomized controlled trials were considered to be high quality, but were downgraded by 1 level (serious) or 2 levels (very serious) for each of the following: limitations of detailed design and execution (risk of bias) (e.g., limitations in randomization, deviations from intended interventions), unexplained heterogeneity or inconsistency of results, indirectness of evidence, imprecision of results, and presence of publication bias.

Self-reported outcomes are the standard in contraceptive research, but they are subject to social desirability bias.^53^ Additionally, intervention and control participants recruited from the same hospital or clinics might have shared intervention contents with each other, resulting in contamination.

### Limitations

It is important to acknowledge that this review only included RCTs and nonrandomized studies to evaluate the effectiveness of mHealth interventions. Other types of evidence may exist and ought to be considered when evaluating the effectiveness of mHealth interventions for family planning. For example, policy makers and other key stakeholders may find equal value from assessing how well an mHealth-mediated family planning program has achieved its goals and outcomes through other types of study designs that blend research with evaluation (e.g., one-group). Another consideration pertains to whether evidence on mHealth interventions conducted in LMICs is disseminated in peer-reviewed outlets (e.g., journals), as noted by Gurman et al.^54^ in their systematic review.

## CONCLUSION AND RECOMMENDATIONS

The use of mobile phones and smartphones in LMICs has proliferated, suggesting mHealth might be a viable tool for delivering interventions aimed at improving family planning outcomes. However, there is insufficient evidence to conclude whether mHealth interventions improve contraceptive uptake in LMICs based on the findings from this review and other systematic reviews.^26^ ^,^ ^28^ Although 3 of 8 studies in this review showed significant improvement in contraceptive outcomes, their effectiveness cannot be linked to specific mHealth features or BCC components.

Moreover, the quality of evidence suggests that improvements in the implementation fidelity and use of behavior change theories are needed for future mHealth family planning interventions in LMICs. Further investigation is warranted to assess and identify which mHealth features, BCC components, and theories, as well as in what specific combinations, will lead to better family planning outcomes and for which specific groups and LMIC locations.

## Notes

**Peer Reviewed**

**First published online:** November 12, 2020
